# Parkinson’s Disease Motor Symptom Progression Slowed with Multisensory Dance Learning over 3-Years: A Preliminary Longitudinal Investigation

**DOI:** 10.3390/brainsci11070895

**Published:** 2021-07-07

**Authors:** Karolina A. Bearss, Joseph F. X. DeSouza

**Affiliations:** 1 Center for Vision Research, York University, Toronto, ON M3J 1P3, Canada; desouza@yorku.ca; 2 Departments of Psychology, York University, Toronto, ON M3J 1P3, Canada; 3 Neuroscience Graduate Diploma Program and Interdisciplinary Studies, York University, Toronto, ON M3J 1P3, Canada; 4 Departments of Biology, York University, Toronto, ON M3J 1P3, Canada; 5 Canadian Action and Perception Network (CAPnet), Multisensory Neuroscience Laboratory, Vision: Science to Applications, York University, Toronto, ON M3J 1P3, Canada

**Keywords:** multisensory therapy, motor symptoms, Parkinson’s disease, neurorehabilitation, longitudinal

## Abstract

Parkinson’s disease (PD) is a neurodegenerative disease that has a fast progression of motor dysfunction within the first 5 years of diagnosis, showing an annual motor rate of decline of the Movement Disorder Society Unified Parkinson’s Disease Rating Scale (MDS-UPDRS) between 5.2 and 8.9 points. We aimed to determine both motor and non-motor PD symptom progression while participating in dance classes once per week over a period of three years. Longitudinal data was assessed for a total of 32 people with PD using MDS-UPDRS scores. Daily motor rate of decline was zero (slope = 0.000146) in PD-Dancers, indicating no motor impairment, whereas the PD-Reference group showed the expected motor decline across three years (*p* < 0.01). Similarly, non-motor aspects of daily living, motor experiences of daily living, and motor complications showed no significant decline. A significant group (PD-Dancers and PD-Reference) by days interaction showed that PD who train once per week have less motor impairment (M = 18.75) than PD-References who do not train (M = 24.61) over time (*p* < 0.05). Training is effective at slowing both motor and non-motor PD symptoms over three years as shown in decreased scores of the MDS-UPDRS.

## 1. Introduction

Parkinson’s disease (PD) is referred to as a movement disorder because of the associated tremors, stiffening or rigidity of movements, slowing of movements (bradykinesia) and postural instability (balance). However, PD also affects many other body symptoms not associated to movement such as anxiety, depression, dementia and mild memory and thinking problems as well as executive dysfunction (ED). The progression of these PD motor [1,2] and non-motor [3,4] symptoms negatively impact function and quality of life (QoL). Studies have shown beneficial effects of gait speed, balance, locomotion and aspects of quality of life from various styles of dance classes: including dance that incorporates ballet, jazz, contemporary, theater and choreography, as well as a well-developed dance curriculum known as Dance for Parkinson’s Disease (DfPD) classes [5,6,7,8,9,10,11]. Dance offers an enjoyable, multidimensional enriched environment where involvement in such a task provides dancers with the necessary tools to enhance balance, coordination, flexibility, imagery, imitation, creativity, rhythm, memory and learning—all of which contribute to improvements in motor symptoms [5,6,7,8,9]. In addition, dance enhances social connection, reduces stress and tension, and boosts confidence and self-esteem leading to an overall improvement in mood [7,12]. In addition, research on dance in PD has shown improvements in patient-caregiver QoL [13] thus we encouraged the caregivers to enroll in the class.

Research on the effects of dance for people with PD (PwPD) has mainly focused on short-term [14,15,16,17,18,19,20] functional outcomes in motor [5,6,9] and non-motor [9,21] symptoms. A few studies in PD have investigated longer intervention periods ranging from six months [20,21], twelve months [22,23,24,25] or as long as two years [26,27]. No research to date has examined how long-term participation in dance impacts disease progression greater than two years. The longest research to date is a 2-year study by McRae et al. (2017), which evaluated QoL, self-efficacy, the effect of DfPD classes on daily activities outside of class and functional mobility in PD participants volunteering in DfPD. They found that DfPD classes positively impacted both social and emotional function outside of the classes, and that motor functioning affects QoL through self-efficacy [26]. Although this study demonstrated the positive influence dance has on social and emotional function in PwPD, it lacked using a motor rating assessment that is most widely applied in PD such as the Movement Disorders Society—Unified Parkinson’s Disease Rating Scale (UPDRS Part III motor scale). In addition, research has yet to show how continuous participation in dance class impacts the progression of PD motor and non-motor symptomology.

A study conducted by Duncan and Earhart (2014) [27] used UPDRS Parts I through III, respectively. The results showed lower scores for all three UPDRS measures at 12- and 24-month follow-up in the five Argentine tango participants in comparison to five PD patients in the control group. To date, Duncan’s (2014) [27] research is the only longitudinal study which utilizes the UPDRS as its assessment tool. Our study is the most up to date longitudinal follow-up seen in this field of research that was last updated by Duncan in 2014. Since Duncan’s (2014) study used the same assessment tools as our current study (all parts of the UPDRS) over a long period of time, thus we are treating Duncan’s (2014) study as a precedent to help shape and guide our current study. With that, our study not only expands the time duration of this line of research to include data for over a three-year period (over one year longer than Duncan’s 2014 study) but it also increased the sample size to sixteen (16) PwPD dance trained participants (an increase of 220% in sample size).

The first aim of this current preliminary report is to evaluate our PD-Dance cohort through an interim period on progression of the motor and non-motor PD symptoms while participating in weekly DfPD classes for over three years. Ultimately, the results of this small-scale preliminary study will allow us to investigate whether using our current outcome measures of all parts of the UPDRS will be feasible to use in a future randomized controlled trial (RCT) leading to our second goal of the study.

To date, research on the progression of cardinal features of PD has shown large variability amongst PD. In a study with average follow-up of approximately six years, Jankovic and Kapadia (2001) assessed overall functional decline in people with PD while on medication, using the UPDRS parts I–III, respectively. Results indicated an annual progression of motor symptoms of 0.704% or total UPDRS III scores of 1.34–1.58, with motor symptoms typically the most affected by PD as the disease progresses [28]. In addition, the authors concluded that age of onset of PD impacts the rate of progression of PD symptoms, such that those with an older age of onset (>57 years) had a more rapid progression of PD in comparison to those with a younger age of onset [28]. Another study exhibited fast progression of motor dysfunction within the first five years, with annual rates of progression of the UPDRS III (motor function) score from 5.2 to 8.9 [29].

In most research investigating progression of PD symptoms, disease progression rates have been defined as the difference between a baseline score and the last score on various measures [29,30]. Our study is the first to follow PwPD over a 3-year period during weekly dance participation, providing additional information regarding the nature of progression of motor and non-motor PD symptoms. Our research goal is to create a long-term neurorehabilitation strategy that combats the symptoms of PD. As such, we utilize a multisensory activity which incorporated the use and stimulation of several sensory modalities in the dance environment including vision, audition, tactile perception, proprioception, kinesthesia, social organization and expression, olfactory, vestibular and balance control—all senses which may influence many of the mood, cognitive, motor and neural challenges faced by people with PD. Over the past four years we have followed and collected data from people with PD while they learned choreography, which is designed to be adaptable to the disease stage and current PD symptoms for those living with PD.

## 2. Materials and Methods

### 2.1. Participants

Participants who had a minimum of two testing sessions between the October 2014 and November 2017 were included in the study. Therefore, a total of sixteen PwPD; mild-severity (M*_H&Y_* = 1.3, SD = 0.9), (*N_males_* = 11, M*_DxYears_* = 5.5, SD = 4.5) agreed to an ongoing, longitudinal, weekly participation consisting of a 1.25-h DfPD class at Canada’s National Ballet School (NBS) and Trinity locations in Toronto, Ontario, over a 3-year period and thus had longitudinal data included in this report. These 16 initial volunteers remained in our study during the course of the staggered 3-year data collection period and thus we had a 0% drop out rate for our study. The ethical protocol was approved by York University and written informed consent was obtained prior to data collection. There are no ethical concerns for this study. Fifteen PD-Dancers provided their age and age at PD onset. Of the sixteen participants, 13 were diagnosed with PD > 57 years of age, where the average age at diagnosis was 63.9 (SD = 11.5). Overall DfPD exercises for each PD dancer were recorded in hours and shown in Table 1. Exercise for this current study was defined as any activity that provides both aerobic and anaerobic movements.

Since the PD non-dance group was impossible to select from our population of PD-Dancers and under the limit of non-exercise related conditions, a reference group, consisting of 16 non-dance PD participants were chosen from a larger PD cohort from the Parkinson’s Progression Marker Initiative (PPMI). It is a longitudinal research project mandated to identify PD markers funded by the Michael J. Fox Foundation for Parkinson’s Research (MJFF) and related funding partners (www.ppmi-info.org/fundingpartners, accessed on 7 July 2021). These 16 PD-Reference group participants were matched on the means of age and gender (*N_Males_* = 11), Hoehn and Yahr (H and Y) score (mild-severity, M*_H&Y_* = 1.6, SD = 0.5) and disease duration to our PD-Dancers group (Table 1), and thus formed our longitudinal PD-Reference non-dance group that would define the baseline standards in our study.

In order to capture weekly exercises for this group, we used a subsection of the Physical Activity Scale for the Elderly (PASE) called Leisure Time Activity. PASE is a reliable and dependable questionnaire used to measure physical activity assessment in older adult populations while relating physical activity to fall and fracture risks as well as gait and balance characteristics, all of which are prominent symptoms of PD. Focusing on questions 4b and 5b, which ask how many hours per week did the subject engage in either ballroom dancing, aerobic dance or both, we are able to conclude that 13 of the PD subjects (3 subjects did not have data for the PASE) did not engage in any form of dance throughout the duration of the study (Table 1).

### 2.2. Ethical Compliance Statement

The study was approved by the Office of Research Ethics (ORE) committee at York University (REB#2013-211 and 2017-296). Prior to any data collection, written informed consent was obtained from each participant.

### 2.3. Measures

UPDRS scores for non-motor aspects of daily living (part I), motor experiences of daily living (part II), motor examination (part III) and motor complications (part IV) were used to assess motor and non-motor PD symptoms. Motor examination was assessed before participation in the 1.25 h, weekly DfPD class, while the remainder UPDRS parts I, II and IV were assessed once after each dance class. Motor assessments were recorded and labeled as non-identifying terms in order to blind our 7 or 8 raters who were trained on scoring the UPDRS using the online training program: a certificate exam developed by The International Parkinson and Movement Disorder Society (MDS). Research has shown that reviewing exercises can improve the reliability of the measures in the UPDRS16.

### 2.4. Procedure

Sixteen subjects trained in a weekly 1.25-h DfPD class for a total of 82,111 [range/subject = 1027 to 10,391] minutes of training. Classes began with live music during the seated warm-up, followed by “barre” work, and ended with moving across the floor; choreography was also learned for an upcoming performance (see Bearss et al., 2017 [9] for dance class details). UPDRS III was videoed and scored by 7–8 MDS-trained experimenters. UPDRS I, II and IV were self-reported on a paper and pen basis and returned the following week at class.

### 2.5. Analysis

Linear mixed effects model analysis allowed us to account for individual variability (*n* = 16) while simultaneously accounting for sixty dance training sessions and was our predefined analysis plan (SPSS, IBM Corp. Released 2016. IBM SPSS Statistics for Windows, Version 24.0. IBM Corp, Armonk, NY, USA). An average slope was generated for each subsection of the UPDRS by calculating the slope of each individual participant across time and then averaging across subjects’ slopes, creating an average slope of each individual participant and the corresponding linear fit which then was compared to a slope of zero.

## 3. Results

**Figure 1:**
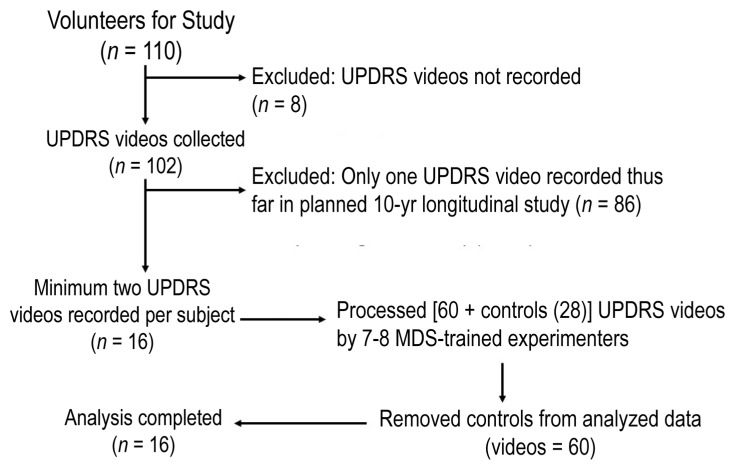
Flow chart of enrolment.

**Figure 2:**
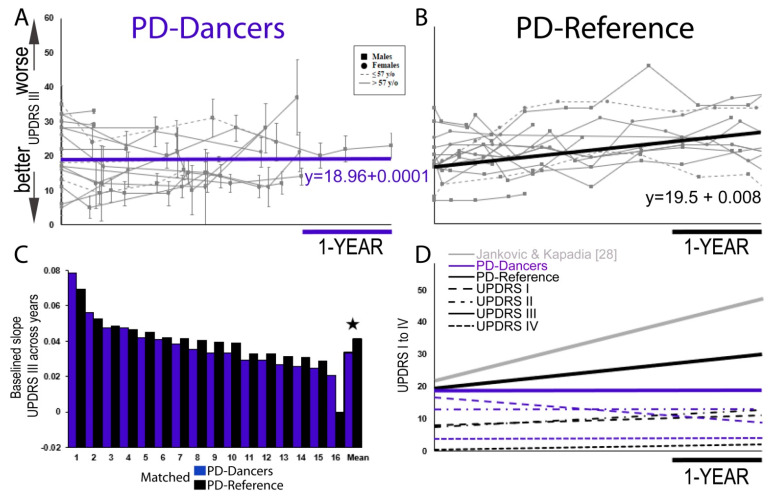
Progression of Parkinson’s disease: PD-Dancer average slopes are indicated by blue color lines and all PD-Reference slopes are represented by black lines. (**A**) PD-Dancer (*n* = 16) scores for UPDRS part III (motor examination) across 3 years. Squares (circles) represent scores for males (females); dashed lines indicate participants ≤ 57 years of age at diagnosis, and solid lines indicate age of diagnosis at >57 years of age. Error bars represent the standard error across each experimenter (7–8) scoring for an individual testing session. Solid black line indicates average slope of 0.000146% rate of decline. (**B**) Matched PD-Reference (*n* = 16) scores for UPDRS part III (motor examination). Solid black line indicates average slope of 0.008% annual motor rate of decline. Same conventions as Figure 2A, except there are no error bars from UPDRS III data since it was rated by one Movement Disorder Society (MDS) experimenter. Only 3 years of data is displayed. (**C**) Baselined individual slopes for all 16 PD-Dancers and 16 PD-References sorted from largest to smallest slopes. ★ *p* < 0.05. (**D**) Summary of all UPDRS I–IV scores. Grey line indicates Jankovic and Kapadia (2001) [28] UPDRS III annual rate of decline.

UPDRS videos were recorded for three years which were then sorted to the 16 subjects who fit our longitudinal criteria of having two sessions (total 60 videos with a mean of 3.75 sessions/subject (range 2–6); Figure 1). As with many neurodegenerative diseases, our PD-Dance group was heavily male gender specific. As such, our PD-Reference group was well balanced in the gender demographic variable to ensure no gender differences would arise, influencing the results. Each subject’s averaged UPDRS III score for each dance session was plotted and lines were drawn for all 16 subjects across all the time points that were recorded (Figure 2A). As noted in Table 1, the total amount of DfPD exercise (in hours) differed across our PD-Dancers within the 3-year data-collection period as not all of the 16 participants were scheduled for consistent data collection within this 3-year time frame. The average slope across all 16 subjects was then computed and plotted (thick blue line in Figure 2A). There is no motor impairment (UPDRS part III) across time (*p* = 0.817) with a daily rate (slope) of 0.000146, which is non-significant from a slope of zero. Surprisingly, non-motor aspects of daily living (I) across time (*p* = 0.329) with a daily rate of –0.0072, motor experiences of daily living (II) across time (*p* = 0.540) with a daily rate of –0.000298, and motor complications (IV) across time (*p* = 0.390) with a daily rate of –0.0000069 also did not show any impairment across time in our dance trained PD group; Figure 2D—see dashed, dotted blue lines).

**Figure 3:**
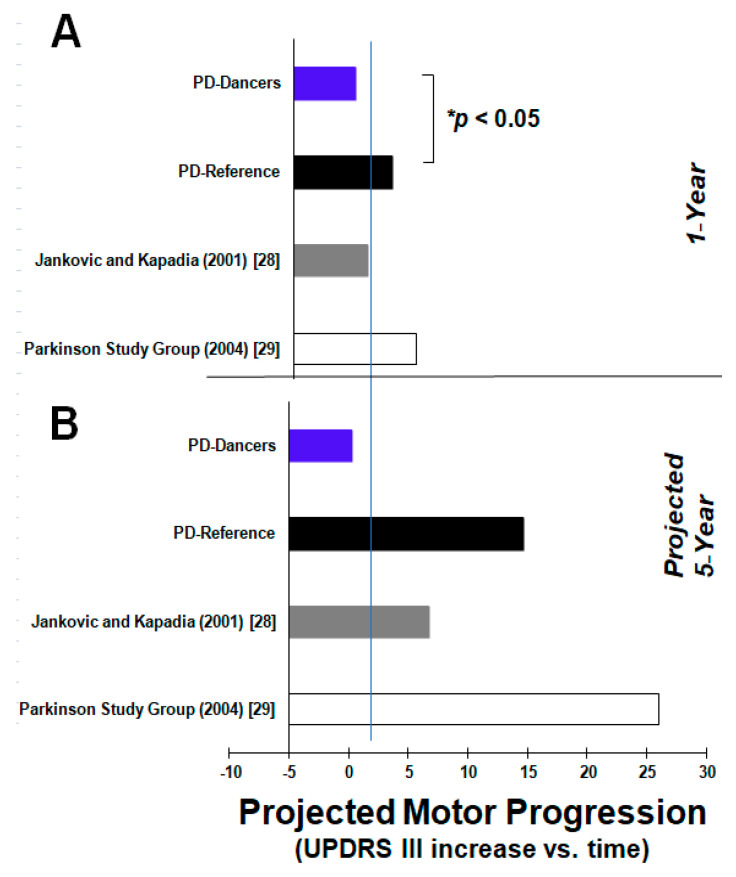
Total annual rate of motor score (UPDRS III) progression across all groups and studies discussed in the text. Based on reported values with Jankovic and Kapadia (2001) [28] the annual rate of progression during the ON-state is quoted. Motor scores after (**A**) 1 year are plotted based on available data and (**B**) 5 years are projected for each group based on current slope measurements.

A significant group (PD-Dancers and PD-Reference) by days interaction showed that PwPD who train weekly have less motor impairment (M = 18.75, SD = 7.82) than PD-Reference who do not train (M = 24.61, SD = 9.67) and over time (*p* < 0.05). To get the motor score change over years, we computed all UPDRS III scores from days into years where we then performed the mixed effects analysis on the GROUP (PD-Dancers and PD-Reference) by years interaction. From this model, we determined that PwPD who train once per week had an overall annual slower rate of change in motor scores when compared to PwPD who do not train (β = −2.93, t = −3.35, *p* < 0.01) (Figure 3). In addition, as expected from the previous literature [31,32,33,34], PD-Reference showed motor impairment (UPDRS III) across time (*p* < 0.01, Figure 2B) with a daily rate (slope) of 0.008. In addition, PD-Reference UPDRS I and II showed disease progression of PD over time (*p* < 0.005) with a daily rate (slope) of 0.0017, and (*p* < 0.01) with a daily rate (slope) of 0.0027 in subjects who did not dance. Whereas PD-Reference UPDRS IV showed no progression (*p* = 0.365) with a daily rate (slope) of 0.0008 (Figure 2D). Figure 2C display’s individual slopes for both groups, PD-Dancers (blue bars) and PD-Reference (black bars), respectively, that were baselined to the lowest slope score in the PD-Reference data set. Mean slopes were plotted at the end of the graph, indicating a significant difference between the two groups where PD-Dancers had less motor impairment than PD patients who do not train in dance (*p* < 0.05) Hedges’ *g* = 0.67 indicating a medium effect.

## 4. Discussion

This study is the first to show that neither the motor nor the non-motor PD symptoms progress in this disease with participation in longitudinal neurorehabilitation training over three years (of our 10-year on-going project). This is markedly different from all previous studies, which showed annual rates of decline for PD increasing at a slope rate of 0.704%/year [28] or 5.2–8.9/year within the first 5 years [29]. Additionally, we confirmed this continual decline in a new cohort—our matched PD non-dance group (PD-Reference), where for the duration of the study these subjects had zero hours of exercise involving dance as measured by questions 4b and 5b of the PASE. Considering demographics, our PD sample had a mean disease duration of 5.54 years (SD = 4.52) which would make our population vulnerable to a rapid symptom decline within the first 5 years [29]. Most importantly, our PD subjects average age at PD diagnosis was 63.93 years (SD = 11.54) and according to Jankovic’s study [28], those who are >57 years of age at disease onset should show the most rapid motor decline. Remarkably, our dancing participants did not demonstrate this disease progression; however, our matched PD-Reference did show this reported PD disease progression. We further modelled our data and computed that after completing 1000 days of training our PD dancers will have a UPDRS III motor score of 19.07 whereas our PD-Reference will score 28.27. Our data further showed that training in dance will slow the rate of PD motor impairment progression, as measured by the UPDRS III, by close to 3 points annually in comparison to our PD subjects who did not train (Figure 3). Since motor PD symptom progression has been shown to be the fastest within the first 5 years [29] of diagnosis, we expanded these motor scores to 5 years and displayed the results across all studies and groups in Figure 3. The results in Figure 3 indicate that training in dance for 1 year will have a 3 point lower UPDRS III score in comparison with no training; these differences in scores increase after 5 years where no training leads to a 15-point higher motor score in comparison with those who do train. These results support previous findings in the literature which indicate fast motor progression within the first 5 years of PD [29]; however, what is of importance here is that this rapid motor progression is not shown with consistent weekly training, and motor impairment progression remains much slower. The reasons for our findings could be due to the additive effects of training, socialization, support and group dynamics that putatively occur within and around the classes [5,6,9,19,30,35,36,37]. Our future studies will continue to examine this cohort with these as dependent measures where possible.

A growing body of evidence demonstrates that high-intensity interval training (HIIT) can serve as an effective alternate to traditional PD exercise programs, inducing similar or even superior physiological adaptations in healthy individuals and diseased populations [38,39]. HIIT is a form of physical exercise that is characterized by brief, intermittent bursts of vigorous activity, interspersed by periods of rest or low-intensity exercise [40]. In our DfPD program, the dance classes are structured with a myriad of factors in mind such as training intensity, speed of rhythm, symptom-specific concerns related to balance, cognition, motor skill, depression and physical confidence, as well as activity duration and movement patterns. The professionally trained teachers incorporate movement from modern, ballet, tap, folk and social dancing, and choreographic repertory to engage participants’ minds and bodies within weekly adapting class structures. With this diverse class structure, the DfPD program can be described as being similar to HIIT dance training, as the classes incorporate both seated dance which provide low-intensity exercise with interspersed upbeat, fast-moving dance styles that provide bursts of vigorous activity. HIIT has been shown to be infinitely variable with the specific physiological adaptations induced by this form of exercise, for instance aerobic capacity (measured by peak VO^2^) and movement initiation time all improved following HIIT intervention [40]. An accumulation of recent research shows that long duration and high intensity training, such as HIIT, may induce neuroplasticity and have neuroprotective effects in PD by increasing serum levels of brain-derived neurotrophic factor (BDNF) in both animal models of PD [32] and PwPD [31]. BDNF is a growth protein that has been shown to be protective against the neurodegeneration observed in PD symptoms [33]. Training in dance can thus lead to increases in BDNF levels which ultimately repair and provide further protection to areas of the brain that are damaged by PD, such as the basal ganglia, i.e., substantia nigra, areas responsible for planning and control of motor movement. This reparative and protective neural restoration may be evidenced by the hindrance of motor and non-motor symptoms displayed in our results. A review of studies that incorporated music and dance indicated the beneficial aspects of using this tool as a form of rehabilitation for people with PD as it improves cadence, speed, gait, balance, and stability while stimulating improvements in both the motor and cognitive symptoms in PD [34,41]. The neuroprotective effects of dance are a potential explanation for these results, other underlying neural mechanisms suggest that regular participation in dance facilitates neural activation of PD impaired sensory-motor areas thus influencing the motor control and improving motor symptoms in PwPD [11].

Our study is the first to also examine changes in UPDRS parts I, II and IV over three years. Our results clearly show that the non-motor aspects of daily living (UPDRS part I), motor experiences of daily living (UPDRS part II) and motor complications (UPDRS part IV) show no significant impairment after three years of training once a week. Again, these results markedly differ from those of Jankovic and Kapadia’s [28], in which annual impairment progressed in individuals who were not participating in weekly training, matching the results we show here in our PD-Reference group.

Research on other non-pharmacological exercise programs [42,43,44] designed to reduce the risk of neurodegeneration in PD have shown motor function improvements; however, these alternate programs seem less efficient at improving clinical symptoms and psychosocial aspects of PD, with only 50% or less of results reporting positive effects [42]. In addition, the impact of physical activity appears to be weaker for both cognitive function and depression in PD [44]. Other forms of dance, such as Argentine tango [16], Irish dancing [20] and PD structured dance classes [37], have shown comparable findings to research on DfPD classes, where both motor and non-motor aspects of PD symptoms improve after participating in dance classes.

Dance intervention studies on PwPD have shown that continuous participation in scheduled dance classes improves balance in PwPD as shown by changes of 3–4 points on the Berg Balance score [45]. A large meta-analysis study conducted on the general population by Asmundson et al. (2013) indicated that exercise programs which last for 16-weeks or more produced the greatest anxiolytic effects, thus duration of exercise not only provides motor improvements but it also provides a protective effect against the development of anxiety in healthy older populations—a non-motor symptom that is seen in many PwPD [46]. In addition to testing exercise and dance’s effects on affect, self-efficacy, gait and attentional dual tasks in seven PwPD, we designed a matched-intensity exercise control task [47] and performed the test a few days before or after dance class in the same subject and measured heart rate and electrodermal activity. Heart rate was the same for both dance and matched-intensity exercise but the dual task showed benefits for the dance over matched-intensity exercise suggesting dance trains additional aspects than just movement sequences [47].

The limitations of this study are that it is a small scale preliminary report that was initially conducted to evaluate feasibility, duration and improve our future study design prior to establishing a full-scale research study with the aim of a future RCT design, and thus, the results presented here are of a pilot project, where the interpretations of the results should be approached with caution. The other limitation to our study, that can be found in all pilot studies, those that are not properly randomized and controlled, there is the issue of selection bias. Following this pilot study, the goal is to design a solid randomized true control trial which will eliminate the issue of any selection bias and the interpretations of the results will thus be warranted.

## 5. Conclusions

Our results indicate positive benefits of weekly training for stopping disease progression of motor and non-motor symptoms of Parkinson’s disease. Previous longitudinal studies [28,29] suggest an annual decline in motor function whereas our cohort shows that the annual motor impairment is drastically reduced. These findings strongly suggest the benefits of dance in people with PD as a supplement to a normal treatment regimen.

## Figures and Tables

**Table 1:** Characteristics of people with Parkinson’s disease (PwPD) in the PD-Dancer and PD-Reference groups.

PD-Dancers					PD-Reference					
Subject	Age	Age Onset	H and Y	Total Hours in DfPD Exercises	Subject	Age	Age Onset	H and Y	PASE Activity (h)	
									Q. 4b (Ballroom Dance)	Q. 5b (Aerobic Dance)
10×	70	67	2	107	3002	68	60	2	0	0
10×	66	64	1	125	3018	61	55	2	0	0
10×	76	73	0	124	3021	64	58	2	0	0
10×	70	66	1	116	3028	76	71	2	0	0
10×	83	82	2	173	3051	72	64	2	0	0
10×	52	37	2	64	3810	67	58	1	-	-
10×	59	50	2	113	3958	76	69	1	-	-
10×	73	70	2	105	3962	69	63	1	-	-
10×	77	77	3	17	4076	72	66	2	0	0
11×	58	58	0	84	40,690	72	65	2	0	0
11×	61	50	1	83	40,693	72	66	1	0	0
12×	68	67	1	122	40,740	69	65	1	0	0
13×	73	71	1	24	40,916	77	65	2	0	0
14×	-	-	1	50	50,175	62	57	1	0	0
15×	77	67	0	35	51,971	66	62	2	0	0
16×	68	60	1	17	57,090	74	72	2	0	0
Mean	68.7	63.9	1.3	85.5		69.8	63.5	1.6	0.0	0.0
SD	8.4	11.5	0.9	45.2		4.9	5.0	0.5	0.0	0.0

## Data Availability

The data is available upon request.
